# How social desirability impacts life satisfaction among Chinese youth: mediators of mental toughness and emotional intelligence

**DOI:** 10.3389/fpsyt.2024.1467804

**Published:** 2024-10-17

**Authors:** Fangyan Lv, Zhanhang Ye, Zicheng Liu, Jing Gan, Jingbin Tan, Run Feng, Burebiya Abudurexiti, Meng Yu, Dingguo Gao

**Affiliations:** ^1^ School of Marxism, Sun Yat-Sen University, Guangzhou, China; ^2^ Department of Psychology and Guangdong Provincial Key Laboratory of Social Cognitive Neuroscience and Mental Health, and Guangdong Provincial Key Laboratory of Brain Function and Disease, Sun Yat-Sen University, Guangzhou, China; ^3^ Faculty Development Center, Guangdong University of Technology, Guangzhou, China; ^4^ Department of Medical Psychology, Guangdong Province Hospital of Chinese People’s Armed Police Forces, Guangzhou, China; ^5^ Department of Psychology, School of Public Health, Southern Medical University, Guangzhou, China

**Keywords:** trait social desirability, mental toughness, emotional intelligence, life satisfaction, young adults

## Abstract

Social desirability has been recognized as a predictor of life satisfaction but it has yet to know the mechanism of this effect. This research aimed to explore the relationship between social desirability and life satisfaction, as well as the mediation effects of mental toughness and emotional intelligence. In Sample 1, we asked 1200 youths (12-24 years old) to complete an online questionnaire measuring social desirability, life satisfaction, mental toughness, and emotional intelligence. Results indicated that social desirability had a direct positive effect on youth’s life satisfaction. In addition, mental toughness and emotional intelligence mediated the relationship between social desirability and life satisfaction, showing a chain role of mental toughness and emotional intelligence. A second sample (n = 750) was then used to verify the above findings, and similar results were found. These findings are consistent with our hypotheses, revealing the mechanisms of social desirability in relation to life satisfaction and the important role of mental toughness and emotional intelligence.

## Introduction

As a multifaceted concept, “social desirability” varies in terminology and understanding when applied across various disciplines. Social desirability consists of two factors: impression management and self-deception (1, 2), which are often used to screen and identify individuals who may be biased in their self-reports in psychological assessments and surveys. Traditionally, it has been defined as the tendency of individuals to respond in a manner that is culturally acceptable and favorable. That is, social desirability is used to describe people who tend to portray themselves in a generally favorable manner (3–6). However, more recent perspectives suggest that social desirability could be considered a personality trait (4, 7–9). Uziel proposed “rethinking social desirability” in an influential review (7), suggesting that people with higher social desirability show greater self-control, particularly in social settings. Since then, researchers have proposed that social desirability is an interpersonal-oriented positive trait (4, 10) and that it is related to personal well-being and interpersonal adjustment (7). Some studies have indicated that social desirability is positively associated with forming and maintaining a good reputation (7), marital relationships, and friendships (11, 12), and showing greater perseverance in solving difficult problems (4).

Recently, social desirability has been found to play a significant positive role in a range of positive psychological and behavioral outcomes for individuals (4, 7–9, 13–18). It has been well documented that social desirability is not only positively linked to a variety of positive psychological and behavioral outcomes (7, 11, 12, 15), but that it is also negatively correlated with both criminal and suicidal tendencies (12, 19–22). Researchers have revealed a variety of positive outcomes associated with social desirability, such as increased creativity (10), greater self-control in a public context (16, 17), more honest behaviors (18), better job performance (14), greater perseverance in solving highly difficult problems, and better prediction of future job performance and adaptation to stress during recruitment (4). Studies have also revealed that self-reported mental health is highly related to social desirability (4, 8, 9, 13). Social desirability is a higher-level, complex integration mental function (23) that focuses on improving mental health and job performance. Thus, social desirability has significant effects on healthy defensive behaviors (4). Although it is mentioned that social desirability has a significant impact on mental health and job performance, and may exert its influence through healthy defensive behaviors, the existing literature may not have fully explored whether the relationship between social desirability and life satisfaction is mediated by other factors. Current research may also not have detailed which specific factors might serve as mediators between social desirability and life satisfaction. This indicates the need for further research into potential mediating variables to verify this relationship and its mediating mechanisms. Therefore, in the current research, we focus on whether the relationship between social desirability and life satisfaction is mediated by mental toughness and emotional intelligence among Chinese youth.

In the era of digital connectivity and social media, the construct of social desirability has become increasingly influential, particularly among youths. This study aims to delve deeply into the impact of social desirability on life satisfaction, with a particular focus on the mediating roles of mental toughness and emotional intelligence. The academic significance of this research lies in its contribution to the existing literature by exploring these constructs within the unique cultural context of China, potentially offering new insights that differ from Western perspectives. Furthermore, this study challenges some existing theoretical assumptions about social desirability. Traditional views may regard social desirability as a construct of response bias, while our research reveals that it may actively influence life satisfaction in a more complex manner through the mediating roles of mental toughness and emotional intelligence. Practically, it can inform interventions aimed at enhancing mental health and well-being in educational and community settings, and also impact policy-making, such as the development of programs to cultivate mental toughness and emotional intelligence. In summary, this study provides a new theoretical perspective and empirical data for understanding the roles of social desirability, mental toughness, and emotional intelligence in adolescent life satisfaction by supplementing and extending existing theories. This not only helps to enrich the theoretical framework of the relevant fields but also has significant implications for guiding practice and improving the mental health and quality of life of Chinese youth.

### Social desirability and young adults’ life satisfaction

Social desirability is a crucial factor in improving young adults’ life satisfaction and is often viewed as a tendency to protect self-esteem by avoiding threatening information and minimizing negative effects (24). This psychological defense mechanism has often been associated with better mental health and life satisfaction (25–27). Previous studies have shown a moderate positive correlation between social desirability and life satisfaction (28), and social desirability is considered an important determinant of life satisfaction (29, 30). Recent evidence also revealed that social desirability is correlated with personal well-being (7, 31). For example, Hitchcott et al. (26) found social desirability is significantly related to higher life satisfaction and fewer depressive symptoms in adults aged 20 to 101 years, consistent with previous research (9, 32, 33). Therefore, the positive effect of social desirability might be an advantageous coping style, which could be a crucial factor in enhancing life satisfaction. Given the positive association between social desirability and life satisfaction, we hypothesized that social desirability will have a positive influence on life satisfaction (hypothesis 1).

### Mental toughness as a potential mediator

Mental toughness is related to life satisfaction (34–37), and is an important individual difference factor for coping with challenges and demonstrating persistence under pressure conditions (37). However, little research has explored the role of mental toughness in the relationship between social desirability and life satisfaction among young adults.

Mental toughness has been theoretically hypothesized to mediate the relationship between social desirability and life satisfaction. It enables some individuals to better persist in many situations, including sports (35, 38–41), education (36, 42–44), the workplace (45, 46); and the military (45, 47, 48). Recent studies have indicated that mental toughness reflects personal resources that are broadly associated with various positive psychological traits such as self-efficacy, optimism, and coping strategies (37, 45, 49). In a longitudinal study conducted by Gerber et al. (50), mental toughness was found to be positively related to life satisfaction. These findings were replicated by Jin and Wang (51), who revealed that mental toughness mediated the relationship between attachment and life satisfaction in a sample of 217 international students.

Although no studies have directly explored the relationship between social desirability and mental toughness, the construct of mental toughness, which has components referring to how one regulates emotions and changes cognition to deal with stressful conditions (52), may be positively correlated with social desirability. Individuals with higher social desirability always favorably portray themselves (53), even under time pressure (54). Meggs et al. (55) in a study of 105 athletes with higher mental toughness had more positive self-concepts. Some studies have also suggested that mental toughness is positively linked to optimism and negatively linked to pessimism (56), extraversion, conscientiousness, neuroticism (57), affiliative and self-enhancing humor styles (58), and the Dark Triads traits (59). From a coping model perspective (60), studies have explored the application of mental toughness to mental health and life satisfaction, indicating that a higher level of mental toughness could predict higher life satisfaction and lower psychological distress (50, 51, 91). Therefore, we hypothesize that mental toughness mediates the relationship between social desirability and life satisfaction (hypothesis 2).

### Emotional intelligence as a potential mediator

Besides mental toughness, emotional intelligence might mediate the relationship between social desirability and life satisfaction. The positive relationship between mental toughness and emotional intelligence indicates that individuals with higher mental toughness have a higher level of emotional intelligence (61). Therefore, emotional intelligence could be proposed as another mechanism to explain the relationship between social desirability and life satisfaction.

It is generally appreciated that individuals with a higher level of emotional intelligence report higher life satisfaction (62–71). Previous research has indicated that emotional intelligence not only directly affects individuals’ life satisfaction, but also plays a mediator role in life satisfaction (70, 72). Several studies have confirmed that emotional intelligence is a significant predictor of life satisfaction (73, 74). One meta-analysis revealed a significant positive correlation (r = 0.32) between emotional intelligence and life satisfaction (34). The essence of emotional intelligence includes perceiving, understanding, harnessing, and managing the complexity and nuances of emotional experiences in the self and others (75). Therefore, we hypothesized that emotional intelligence would play a significant role in the relationship between social desirability and life satisfaction (hypothesis 3).

### The chain mediation effect of mental toughness and emotional intelligence

Mental toughness, which is related to perseverance and success in performance domains, could be a stress buffer for people (76). Emotional intelligence, which influences emotion perception and emotion control and management can promote the mental toughness of athletes. Previous studies found that both mental toughness and emotional intelligence worked together to help athletes cope with stress and improve their performance (77). Studies also found that emotional intelligence was a significant predictor of mental toughness (78). Contrarily, some studies found that mental toughness was a predictor of emotional intelligence (Gucciardi et al., 2008; 61). Given the interaction of these two variables, we propose hypothesis 4 based on previous studies (61). Thus, we hypothesize that mental toughness and emotional intelligence will play a chain mediation role in the relationship between trait social desirability and life satisfaction (hypothesis 4).

### The current study

**Figure 1:**
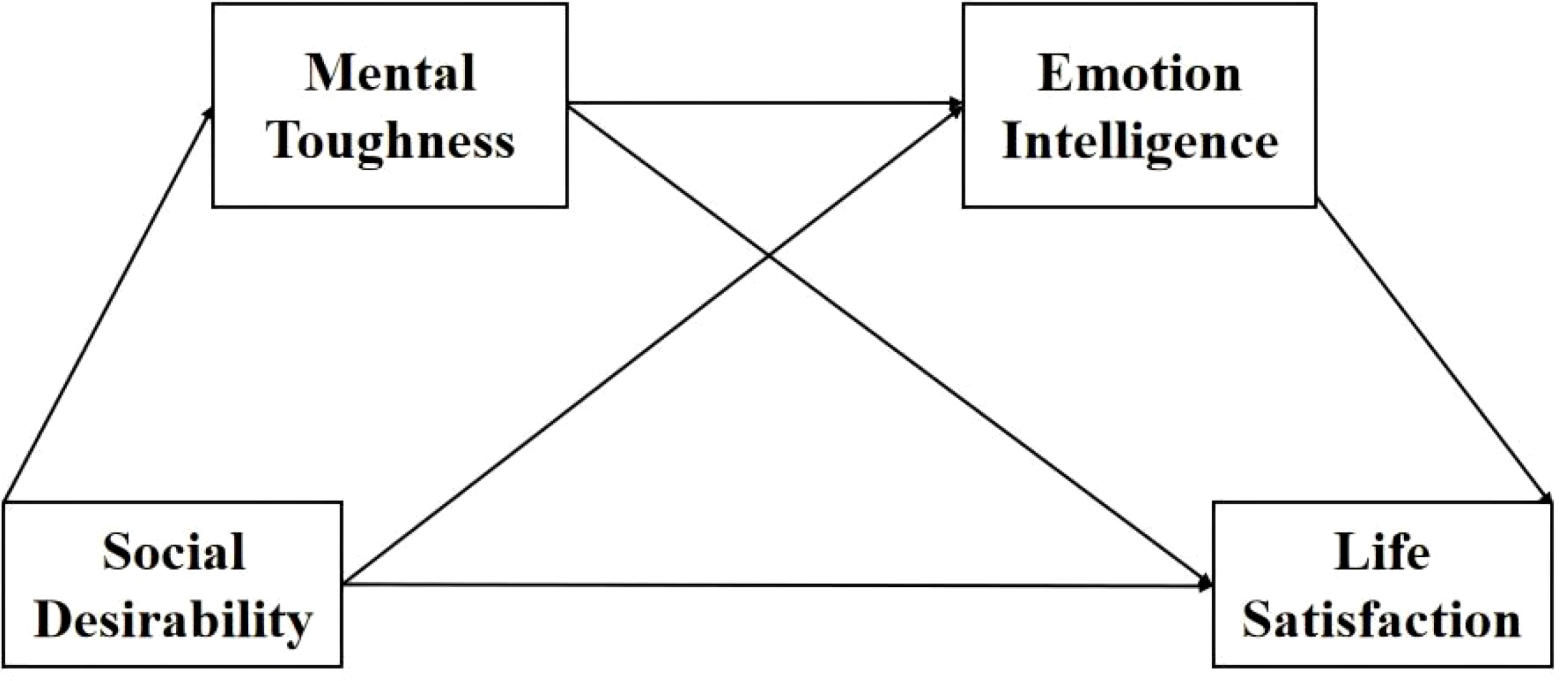
The hypothetical structure model.

To further explore the impact of social desirability on youth’s life satisfaction, the current study has three aims. First, we aim to examine the direct effect of social desirability on life satisfaction. Second, we seek to explore the mediating role of mental toughness and emotional intelligence. Third, we intend to explore whether social desirability predicts life satisfaction through the chain-mediating effect of mental toughness and emotional intelligence. Thus, in the present research, we hypothesized (1) social desirability will have a positive influence on life satisfaction, (2) mental toughness mediates the relationship between social desirability and life satisfaction, (3) emotional intelligence mediates the relationship between social desirability and life satisfaction, mental toughness and emotional intelligence play a chain mediation role in the relationship between trait social desirability and life satisfaction (See **Figure 1**). To examine these objectives, we used two independent samples of Chinese college students. In Sample 1, we conducted a multiple mediation analysis to test the mediation effect of mental toughness and emotional intelligence on the relationship between social desirability and life satisfaction. In Sample 2, we repeated the same procedures on another college student sample.

## Materials and methods

### Participants and procedures

Sample 1 consisted of 1,200 Chinese adolescent and young adult participants enrolled at one middle school, two high schools, and two universities in Guangdong. Participants were aged from 12 to 24 years (*M* = 19.36, *SD* = 2.21), and they received compensation upon completing the survey. Sample 2, the sample consisted of 750 Chinese adolescent and young adult participants enrolled at one middle school, one high school, and two universities in Guangdong. Participants were aged from 12 to 24 years (*M* = 19.19, *SD* = 2.58), and they received compensation upon completing the survey. All data were collected by a web-based survey designed by Wenjuanxing (in Chinese). Informed consent was obtained at the start of the survey. It was made clear to the participants that they could withdraw from the survey at any time by closing their browser without having their responses registered. Questionnaire completion was self-paced. The study was approved by the author’s Institutional Review Board. The participants completed all measures anonymously to alleviate their anxiety about disclosing individual information and to ensure the validity of the survey. The survey took about 15 minutes to complete.

### Measures

#### Social desirability

Social desirability was measured with the Social Desirability Scale-17 (SDS-17; 79), which consists of 16 true–false items (e.g., “I will never live off other people”), and is an updated version of the MCSDS (80). It has been validated in the United States and Germany (81). The Cronbach’s α in the present sample was 0.68 for the SDS-17 total score. Higher scores indicate a stronger tendency to present oneself in a desirable light.

#### Life satisfaction

The Satisfaction with Life Scale (SWLS; 82) was used to measure of global life satisfaction. The SWLS consists of five items (e.g., “I am satisfied with my life”) which were rated on a seven-point Likert-type scale (1= strongly disagree, 7= strongly disagree). A Chinese translation of the SWLS demonstrated adequate reliability and validity (83). The Cronbach’s α of the scale was 0.87 in the present sample.

#### Mental toughness

We assessed mental toughness using a Chinese version of the 27-item Hardiness Scale (84). The Hardiness Scale was developed according to the theory of hardiness/mental toughness and Chinese social culture. It has adequate reliability and validity and is adapted for testing people’s personality traits of mental toughness. Responses are rated on a four-point Likert scale (1= strongly agree, 4= strongly disagree). The Cronbach’s α of the scale was 0.94 in the present sample.

#### Emotional intelligence

Emotional intelligence was measured using the Wong Law Emotional Intelligence Scale (85) which is a 16-item self-report measurement with four sub-dimensions (self-emotion appraisal, appraisal of others’ emotions, use of emotion, and regulation of emotion). The items are scored on a seven-point scale (1 = very strongly disagree, 4 = neutral, 7 = very strongly agree). The Chinese version demonstrates adequate reliability and validity. The Cronbach’s α for the scale was 0.92.

#### Control variables

According to previous research, age, and gender may have an effect on life satisfaction (86, 87), so we controlled for age and gender as covariates in the present study.

The same measures related to social desirability, mental toughness, emotional intelligence, and life satisfaction were used in Sample 2. The Cronbach’s alphas for measures related to social desirability, mental toughness, emotional intelligence and life satisfaction in this study were 0.66, 0.96, 0.94, and 0.90, respectively.

### Analyses

Descriptive analyses, correlations, and mediation analyses were conducted (88). In particular, considering the non-normal distribution of the data, bias-corrected confidence intervals (CI; 95%) based on 5000 bootstrap replications were used to determine the significance of the indirect effect (c’ path). CIs that did not contain zero would indicate significant mediation effects (88).

According to the power tables for mediation analysis (89), supposing a medium effect size (0.39) for the mediation effect (αβ), a minimum sample of 71 participants was required to achieve a minimum statistical power of 0.80. Therefore, the sample size in the study was more than adequate and had a statistical power of over 0.95. **Table 1** provides information on the demographic characteristics of the Sample 1. The same analysis method was used for Sample 2.

**Table 1:** Demographic characteristics of Sample1.

	N (%)	M (SD)	Range (years)
**Age**		19.36 (2.21)	12-24
**Male**	561 (46.75%)	19.43 (2.19)	12-24
**Female**	639 (53.25%)	19.30 (2.23)	13-24
**Total**	1200 (100%)	19.36 (2.21)	12-24

N =1200.

## Results

### Common method bias test

We conducted the common method basis test using the Harman single-factor method to include all items in the self-report scales of social desirability, mental toughness, emotional intelligence, and life satisfaction using exploratory factor analysis. Only 29.17% of the variance was explained by the largest factor, which was less than the critical value of 40%. The results indicated that there was no significant common method bias in the current study.

### Correlation analysis

The results from the Pearson correlation analysis indicated that social desirability, mental toughness, emotional intelligence, and life satisfaction, all shared a significantly positive correlation. **Table 2** provides the results of the correlation analysis.

**Table 2:** Descriptive statistics and correlation analysis for all variables.

Variables	M	SD	1	2	3	4	5	6
1 Social desirability	10.38	3.07	_—_					
2 Mental toughness	70.23	14.90	0.32^***^	_—_				
3 Emotional intelligence	81.46	14.34	0.35^***^	0.64^***^	_—_			
4 Life satisfaction	20.70	6.89	0.30^***^	0.43^***^	0.43^***^	_—_		
5 Age	19.36	2.21	-0.04	-0.05	-0.03	-0.04	_—_	
6. Gender			0.04	-0.12^**^	-0.06^*^	0.07^**^	-0.03	_—_

^***^p <.001, **p <.01, *p <.05.

Our findings suggest that greater levels of social desirability were positively associated with higher levels of life satisfaction (*r* = 0.30, *p* < 0.001), and that both social desirability and life satisfaction were separately positively associated with mental toughness (*r* = 0.32, *p* < 0.001; *r* = 0.43, *p* < 0.001) and emotional intelligence (*r* = 0.35, *p* < 0.001; *r* = 0.43, *p* < 0.001). Mental toughness was significantly correlated with emotional intelligence (*r* = 0.64, *p* < 0.001).

### Single mediation analysis

A single mediation analysis was first conducted to examine the mediating role of both mental toughness and emotional intelligence in the relationship between social desirability and life satisfaction after controlling for age and gender as covariates. As we hypothesized, mental toughness exerted a significant mediation effect on the relationship (estimate = 0.28, 95% CI = [0.22, 0.35]). Additionally, emotional intelligence also exerted a significantly mediation effect on the relationship (estimate = 0.30, 95% CI = [0.23, 0.37]).

### Multiple mediation analysis

**Figure 2:**
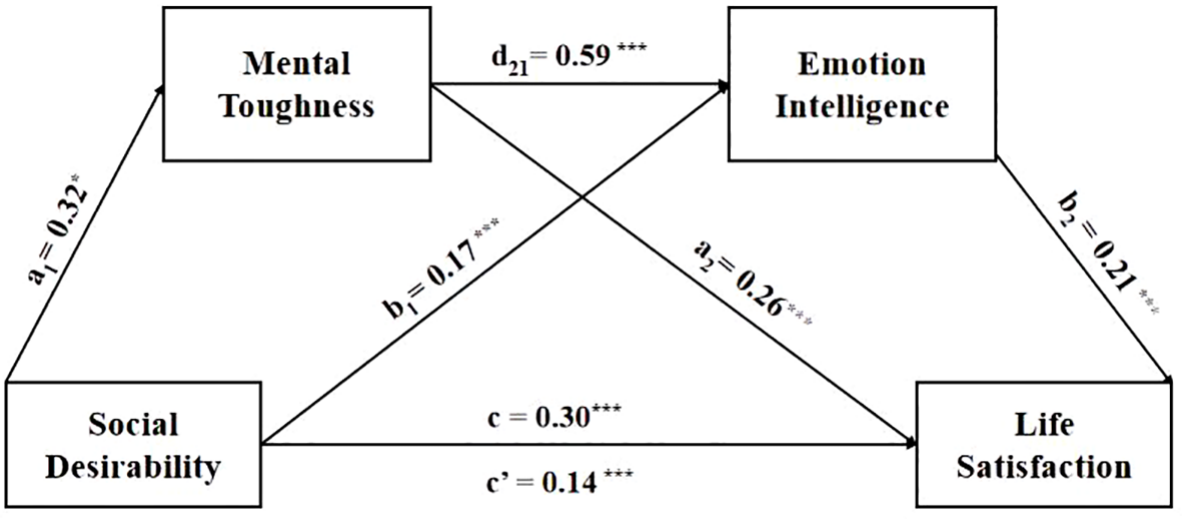
Path diagram of mediator model based on Sample 1. *p <.05,***p <.001.

As shown in **Table 3**, we explored the mediation effect of mental toughness and emotional intelligence on the relationship between social desirability and life satisfaction using a chain mediation analysis in PROCESS (88). As predicted, social desirability was significantly associated with life satisfaction, b = 0.30, p < 0.001, 95% CI = [0.54, 0.79] (Model 1). The results showed that social desirability significantly positively predicted mental toughness, b= 0.32, p < 0.001, 95% CI = [1.32, 1.83] (Model 2) (see **Figure 2** a1 path). Model 3 showed that social desirability positively and significantly predicted emotional intelligence significantly, b = 0.17, p < 0.001, 95% CI = [0.56, 0.98] (see **Figure 1** a2 path), and mental toughness was positively associated with emotional intelligence, b = 0.59, p < 0.001, 95% CI = [0.52, 0.61] (see **Figure 2** b21 path). Furthermore, mental toughness (b = 0.26, p < 0.001, 95% CI = [0.09, 0.15] (see **Figure 1** b1 path) and emotional intelligence (b = 0.22, p < 0.001, 95% CI = [0.07, 0.13] (see **Figure 1** b2 path) positively predicted life satisfaction, Meanwhile the direct effect of social desirability on life satisfaction was significant (Model 4), b= 0.30, p< 0.001, 95% CI = [0.18, 0.42] (see **Figure 1** C’ path). The results of the mediation analysis confirmed our hypotheses. The results indicated that the overall model explained 66.54% of the variance in life satisfaction, the standardized regression coefficient (β) for each path was significant, ps < 0.001, and the overall explanation rate of social desirability on life satisfaction was 45.54%. With the mediators controlled, the direct link of social desirability with life satisfaction remained significant, β = 0.30, p < 0.001, revealing that mental toughness and emotional intelligence had partial mediation effects on the relationship.

**Table 3:** Multiple regression of the mediation effect.

	Model1 (LS)		Model 2 (MT)		Model 3 (EI)		Model 4 (LS)	
	*β*	*t*	*β*	*t*	*β*	*t*	*β*	*t*
Gender	0.05^*^	1.99	-0.14^***^	-5.01	0.01	0.24	0.11^***^	4.22
Age	-0.03	-0.97	-0.04^***^	-1.43	0.01	0.10	-0.01	- 0.48
SD	0.30^***^	10.78	0.32^***^	11.95	0.16 ^***^	7.16	0.14^***^	5.00
MT					0.59^***^	25.32	0.26^***^	7.97
EI							0.22^***^	6.45
R	0.31		0.35		0.66		0.50	
R^2^	0.09		0.12		0.43		0.25	
F	41.42^***^		55.41^***^		229.31^***^		81.10^***^	

^*^p <.05, ^**^p <.01, ^***^p <.001.

As presented in **Table 4**, the bootstrap analysis found that the 95% BootCIs of mental toughness (estimate = 0.16, 95% CI = [0.06, 0.11]) and emotional intelligence (estimate = 0.04, 95% CI = [0.02, 0.05]) did not contain zero, thereby indicating that the mediation effects of mental toughness and emotional intelligence in the relationship between social desirability and life satisfaction were significant. In addition, a path from social desirability to life satisfaction through mental toughness and emotional intelligence remained significant (estimate = 0.04, 95% CI = [0.03, 0.06]), revealing that the link between social desirability with life satisfaction could be mediated by the serial path of mental toughness and emotional intelligence (see **Figure 2**).

**Table 4:** Standardized indirect effects and confidence estimates for Bootstrap estimates in Sample 1.

Model pathways	Estimates	BootSE	95% BootCI	
			Lower	Upper
Total effect	0.30^a^	0.06	0.54	0.79
Direct effect	0.14^a^	0.06	0.18	0.42
SD→MT→LS	0.09^a^	0.01	0.06	0.11
SD→EI→LS	0.03^a^	0.01	0.02	0.05
SD→MT→EI→LS	0.04^a^	0.01	0.03	0.06

CI, confidence interval; SD, social desirability; MT, mental toughness; EI, emotional intelligence; LS, life satisfaction.

a Empirical 95% confidence interval does not overlap with zero.

The same method of analysis was used on the data obtained from Sample 2. **Table 5** presents the descriptive statistics and correlations for all variables. The results indicated that social desirability, mental toughness, emotional intelligence, and life satisfaction had a significant positive correlation with each other (ps <.01). In Sample 2, we conducted a multiple mediation analysis to examine the mediating role of mental toughness and emotional intelligence in the relationship between social desirability and life satisfaction after controlling for age and gender. The results indicated that the mediation effects of mental toughness (estimate = 0.20, 95% CI = [0.12, 0.28]) and emotional intelligence (estimate = 0.06, 95% CI = [0.02, 0.11]) in the relationship between social desirability and life satisfaction were significant. Furthermore, a path from social desirability to life satisfaction through mental toughness and emotional intelligence remained significant (estimate = 0.11, 95% CI = [0.06, 0.16]), revealing that the relationship between social desirability and life satisfaction could be mediated by the chain path of mental toughness and emotional intelligence.

**Table 5:** Descriptive statistics and correlations for all variables in Sample 2.

Variables	*M*	*SD*	1	2	3	4	5	6
1 Social desirability	11.00	2.97	_—_					
2 Mental toughness	70.85	15.56	0.33^***^	_—_				
3 Emotional intelligence	82.16	15.07	0.32^***^	0.66^***^	_—_			
4 Life satisfaction	21.82	6.87	0.27^***^	0.43^***^	0.42^***^	_—_		
5 Age	19.19	2.59	-0.03	-0.04	-0.03	0.03	_—_	
6. Gender			0.06	-0.13^**^	-0.05	0.02	0.04	_—_

^***^p <.001, ^**^p <.01, ^*^P <.05.

Lastly, we also conducted an exploratory moderation analysis to test whether mental toughness and emotional intelligence may moderate the relationship between social desirability and life satisfaction. The results indicated that mental toughness and emotional intelligence did not moderate this relationship between social desirability and life satisfaction (see **Supplementary Materials** for Samples 1 and 2).

## Discussion

The present study primarily investigated the direct effect of social desirability on life satisfaction and the indirect effects of mental toughness and emotional intelligence. The results indicated that social desirability not only directly impacts youth’s life satisfaction but also has indirect effects through the mediators of mental toughness and emotional intelligence.

The current research revealed that social desirability had a significant positive effect on life satisfaction. These findings are consistent with previous research on the relationship between social desirability and life satisfaction (7, 26, 31). Previous research indicated that higher social desirability predicts higher life satisfaction (7, 25–31). Even after controlling for age and gender, social desirability was an important personal trait for improving young adults’ life satisfaction.

The present research revealed that mental toughness played a mediating role in the relationship between social desirability and life satisfaction, which extends previous findings. Mental toughness is often viewed as a resilience resource or factor because both concepts involve one’s capacity to cope with stressful situations and the ability to bounce back from adversity (51). However, there are differences among them: mental toughness focuses on one’s ability to deal with stress and hardship (60) while resilience considers one’s general attitudes and beliefs regarding stressful situations (51). Therefore, a positive association between social desirability and mental toughness suggests that mentally tough individuals tend to cope with challenges and to persist under hardship (37), while individuals with high social desirability cope with stressful situations more effectively and have more perseverance in solving difficult problems and enduring boredom (4). Another important composite of mental toughness was confidence in ability and interpersonal skills (90). Mentally tough individuals are able to maintain high levels of control and confidence, allowing them to push themselves forward in social settings and leading to better life satisfaction. Therefore, there is a clear relationship between social desirability and mental toughness.

From a theoretical perspective, the current findings provide further evidence to support the notion that social desirability is associated with life satisfaction and that interpersonal adjustment could represent a vital bridging concept between the constructs of social desirability and mental toughness. In addition, the findings support the previously made assumption that individuals with a higher level of mental toughness might have more life satisfaction (35–37). On the practical side, the significant mediation effect provides further support to the findings from several previous studies which tested the effectiveness of mental toughness training for young adults. Such training has been found to lead to decreased depressive symptoms (91, 92), and better life satisfaction and mental health (93, 94). Recent studies on youth mental health have begun to pay more attention to mental toughness (35, 37, 38). Given that the World Health Organization conducted the World Mental Health International College Student project, which focused on correlates of common mental health disorders among first-year college students (95), training in mental toughness might be an effective way of improving young adults’ mental health and school performance. Our findings may also support the view that training young adults so that they develop a higher mental toughness may lead to better outcomes.

The current research revealed that emotional intelligence played a mediating role in the relationship between social desirability and life satisfaction. People with high emotional intelligence often have better emotional regulation abilities, allowing them to manage negative emotions such as anxiety and depression more effectively. Previous research confirmed that emotional intelligence is a significant predictor of life satisfaction (73, 74). People with high levels of emotional intelligence contribute to maintaining a high level of life satisfaction (73). Our results are also consistent with previous studies which indicated that emotional intelligence acted as a mediator role in life satisfaction (70–72). One possible reason for this is that emotional intelligence, as a psychological ability, may act as a bridge between social desirability and life satisfaction by influencing an individual’s social interactions, emotional experiences, and decision-making processes. However, this mediation effect may vary due to individual differences, cultural backgrounds, and specific situations. This can help individuals to gain more social approval because emotionally stable individuals are generally more likable. They are usually more adaptable to different social environments and situations. This adaptability may make them more successful in social interactions, thereby increasing their life satisfaction. In addition, our results extended previous studies by revealing that mental toughness and emotional intelligence may make independent contributions to the association in the general young adult population using the multiple mediation model. It would also help contribute to knowledge in the field of social desirability and life satisfaction.

We found that the serial mediation effect of mental toughness → emotional intelligence played an important role in the relationship between social desirability and life satisfaction. This study was the first to explore the potential mechanism linking social desirability and life satisfaction with mental toughness and emotional intelligence. That is, individuals with higher social desirability are expected to display higher mental toughness, and individuals with greater mental toughness would have higher emotional intelligence, which may lead to higher life satisfaction. Taken together, the present results showed that the serial mediation effect of mental toughness→ emotional intelligence could act as a mechanism in the relationship between social desirability with life satisfaction.

Given the replication crisis declared in the region of psychology (96, 97), we conducted another study using an independent sample to test if our results were repeatable. We found that all the results remained significant in Sample 2. The mediation effect of mental toughness and emotional intelligence on the relationship between social desirability and life satisfaction found in Sample 1 was confirmed. Therefore, our findings were found to be stable and repeatable.

The study also has a few limitations. First, the sample in this study consisted of a limited number of Chinese participants aged 12-24 years old, so it is not appropriate to apply the findings to all other young adults. Second, the data used in this study came from only one cultural context. The generalization of the results is limited and the results should not be directly applied to other countries of different cultures. Comparative studies could reveal cultural nuances in how social desirability impacts life satisfaction and whether the mediating roles of mental toughness and emotional intelligence are consistent across cultures. Third, our results were based on a cross-sectional design, which means that we cannot infer causality or the direction of the relationships observed. More tests are needed to further confirm the findings. Given the cross-sectional nature of this research, future studies could benefit from a longitudinal design to examine the causal relationships over time between social desirability, mental toughness, emotional intelligence, and life satisfaction. This approach would help to determine whether changes in one variable precede and predict changes in another. Additionally, the reliance on self-report measures could introduce biases such as social desirability bias, where participants may provide answers that they believe will be viewed favorably by others. Future research could benefit from incorporating objective measures or utilizing experimental designs to mitigate these potential biases. Lastly, the sample size, while sufficient for this preliminary study, may not be large enough to detect smaller effect sizes or to allow for subgroup analyses. A larger and more diverse sample could enhance the external validity of the study and provide a more comprehensive understanding of the phenomena under investigation. This study identified mental toughness and emotional intelligence as key mediators. Future research could explore other potential mediators or moderators, such as personality traits, social support, or coping strategies, that may influence the relationship between social desirability and life satisfaction. By addressing these areas, future research can build upon the current study’s findings, deepen our understanding of the underlying mechanisms, and contribute to the development of effective strategies for promoting life satisfaction among youth.

## Conclusions

The current study indicates that social desirability directly or indirectly impacts life satisfaction through the mediating role of mental toughness and emotional intelligence. It provides a new theoretical framework for researchers to explore the mediation effects of mental toughness and emotional intelligence in the relationship between social desirability and life satisfaction. Future studies could explore this area further to validate and extend the study findings. The findings revealed the probable underlying mechanisms of the effect of social desirability on life satisfaction which may provide us with more important information on how to improve young adults’ life satisfaction.

## Data Availability

The raw data supporting the conclusions of this article will be made available by the authors, without undue reservation.
