# Supplementary material for: How social desirability impacts life satisfaction among Chinese youth: mediators of mental toughness and emotional intelligence
Source: Front Psychiatry. 2024 Oct 17;15:1467804. doi: 10.3389/fpsyt.2024.1467804 (PMC11524859; doi:10.3389/fpsyt.2024.1467804)
Supplement: Supplementary file 1 [file Table1.docx]

**Supplementary Materials**

**Supplementary analyses.**

1. Mediation analysis in Sample 1
2. The mediation of mental toughness in the relationship between social desirability and life satisfaction was not significant (*p* > 0.05). The bootstrap analysis found that the 95% BootCIs of mental toughness (estimate = 0.003, 95% CI = [-0.01, 0.01]) did contain zero, thereby indicating that the moderating effects of mental toughness in the relationship of social desirability with life satisfaction were not significant.

Table 1. Multiple regression of the moderation effect.

| Predictors | Model1  （Life satisfaction） | |
| --- | --- | --- |
|  | *b* | *t* |
| Social desirability | 0.39^***^ | 6.39 |
| Mental toughness | 0.18^***^ | 14.30 |
| Social desirability × Mental toughness | 0.003 | 0.86 |
| R^2^ | 0.48 | |
| F | 70.51^***^ | |

*Note.* ^*^ *p* < .05, ^**^ *p* < .01, ^***^ *p* < .001.

1. The moderation of emotional intelligence in the relationship between social desirability and life satisfaction was not significant (*p* > 0.05). The bootstrap analysis found that the 95% BootCIs of EI (estimate = 0.004, 95% CI = [-0.01, 0.01]) did contain zero, thereby indicating that the moderating effects of emotional intelligence in the relationship of social desirability with life satisfaction were not significant.

Table 2. Multiple regression of the moderation effect.

| Predictors | Model1  （Life satisfaction） | |
| --- | --- | --- |
|  | *b* | *t* |
| Social desirability | 0.38^***^ | 6.08 |
| Emotion intelligence | 0.18^***^ | 13.44 |
| Social desirability × emotional intelligence | 0.01 | 1.08 |
| R^2^ | 0.22 | |
| F | 65.24^***^ | |

*Note.* ^*^ *p* < 0.05, ^**^ *p* < 0.01, ^***^ *p* < 0.001.

1. Mediation analysis in sample 2

The mediation of mental toughness in the relationship between social desirability and life satisfaction was not significant (*p* > 0.05). The bootstrap analysis found that the 95% BootCIs of mental toughness (estimate = -0.001, 95% CI = [- 0.01, 0.01]) did contain zero, thereby indicating that the moderating effects of mental toughness in the relationship of social desirability with life satisfaction were not significant.

Table 3. Multiple regression of the moderation effect.

| Predictors | Model1  （Life satisfaction） | |
| --- | --- | --- |
|  | *b* | *t* |
| Social desirability | 0.32^***^ | 3.93 |
| Mental toughness | 0.17^***^ | 11.20 |
| Social desirability × mental toughness | -0.001 | - 0.11 |
| R^2^ | 0.21 | |
| F | 38.77^***^ | |

*Note.* ^*^ *p* < 0.05, ^**^ *p* < 0.01, ^***^ *p* < 0.001.

1. The mediation of emotional intelligence in the relationship between social desirability and life satisfaction was not significant (*p* > 0.05). The bootstrap analysis found that the 95% BootCIs of emotion intelligence (estimate = 0.001, 95% CI = [- 0.01, 0.01]) did contain zero, thereby indicating that the moderating effects of emotion intelligence in the relationship of social desirability with life satisfaction were not significant.

Table 4. Multiple regression of the mediation effect.

| Predictors | Model1  （Life satisfaction） | |
| --- | --- | --- |
|  | *b* | *t* |
| Social desirability | 0.34 ^***^ | 4.16 |
| Emotion intelligence | 0.17 ^***^ | 10.85 |
| Social desirability × emotional intelligence | 0.001 | 0.06 |
| R^2^ | 0.20 | |
| F | 37.07^***^ | |

*Note.* ^*^ *p* < 0.05, ^**^ *p* < 0.01, ^***^ *p* < 0.001.
